# *Otx2* mRNA expression is downregulated following traumatic brain injury in zebra finches

**DOI:** 10.3389/fncir.2025.1591983

**Published:** 2025-06-06

**Authors:** Adam Talwalkar, Kelli A. Duncan

**Affiliations:** ^1^Program in Biochemistry, Vassar College, Poughkeepsie, NY, United States; ^2^Department of Biology, Program in Neuroscience and Behavior, Vassar College, Poughkeepsie, NY, United States

**Keywords:** avian, neuroprotection, injury, homeoprotein, *Otx2* gene

## Abstract

Traumatic brain injury (TBI) induces a wide range of neurodegenerative symptoms, yet effective treatment strategies remain limited. Emerging evidence suggests that post-TBI recovery recapitulates aspects of early brain development, highlighting the potential for developmental molecular mechanisms to inform therapeutic interventions. The transcription factor *Otx2* is critical for early brain and sensory organ development, as well as the maintenance of retinal and neural function in adulthood. Notably, the transfer of *Otx2* homeoprotein into parvalbumin-expressing (PV+) GABAergic interneurons is essential for opening and closing critical periods of plasticity across vertebrates. Here, we investigate the acute regulation of *Otx2* mRNA following TBI in adult zebra finches (ZF) to evaluate its potential as a target for future study and therapeutic manipulation in neural repair. Adult ZFs sustained unilateral hemispheric brain injuries, and qPCR was used to quantify *Otx2* mRNA expression at 24 hours and 1 week post-injury in both males and females. Our findings reveal a significant downregulation of *Otx2* mRNA expression following injury, highlighting *Otx2* as a potential target for further investigation and manipulation. These results provide insight into the molecular response to brain injury and suggest a potential link between developmental pathways and post-injury plasticity.

## 1 Introduction

Traumatic brain injury (TBI) is a significant public health burden worldwide (Dewan et al., 2019; Flanagan, 2015) and remains one of the leading causes of injury-related death and disability (Maas et al., 2008). TBIs can result in substantial long-term health consequences, including increased risks of developing Alzheimer’s Disease, Parkinson’s Disease, chronic traumatic encephalopathy, and nearly doubling the risk of suicide (Bielanin et al., 2023). Despite this prevalence and burden, effective treatment options remain limited (Watson et al., 2016). While there have been few advances in clinical management, extensive research has focused on identifying therapeutic targets with potential for clinical application (Tabet et al., 2022; Bruhns et al., 2022; Kim et al., 2022; Song et al., 2023).

For decades, researchers have noted parallels between the stages of normal neuronal development and recovery from brain injury (Cramer and Chopp, 2000). Specifically, motor function recovery across vertebrates mirrors the developmental processes of those functions (Cramer and Chopp, 2000; Berenbaum and Hines, 1992; Teitelbaum et al., 1969; Wolgin et al., 1980). Post-injury plasticity involves molecular and cellular events akin to those occurring during normal brain development (Nudo, 2003; Nudo, 2013). Neurons undergo similar stages of growth and reorganization during recovery, suggesting that targeting developmental pathways could enhance therapeutic outcomes. We propose that effective treatments for brain injury should activate these normal developmental processes to promote recovery. Studying neuroplasticity during early recovery phases may yield valuable insights into the brain’s restorative mechanisms (Song et al., 2023; Cramer and Chopp, 2000).

Songbirds provide a valuable model for studying the connections between development and recovery from injury. The songbird brain contains a specialized system of interconnected nuclei responsible for singing and song learning, known as the song system. Estrogens play a critical role in the development and sexual differentiation of song control nuclei in songbirds by influencing neuronal growth and connectivity, particularly during early life stages (Gurney and Konishi, 1980; Schlinger and Arnold, 1992; Cornil et al., 2006). Juvenile songbirds learn to sing during a sensitive period involving two sequential phases—sensory and sensorimotor learning—both of which depend on auditory experience (Mooney, 2009). This naturally occurring framework provides a model for investigating whether developmental organizational patterns re-emerge after injury. Notably, sites of neuronal recovery extend beyond the confines of the song system, suggesting the involvement of broader, more robust regulatory mechanisms. Previous work has identified recovery-related activity in regions outside the song system, revealing striking similarities in the underlying mechanisms (Wynne et al., 2008; Duncan and Saldanha, 2011). Additionally, recent research has identified genes undergoing epigenetic regulation during song learning, offering promising targets for understanding recovery processes in the adult brain (Diddens et al., 2021). For this report, we will focus on the role of orthodenticle homeobox 2 *Otx2*, which has been previously identified during song nuclei development (Diddens et al., 2021) and among vertebrate development in general as it relates to coordination of A-P and D-V patterning by regulating the expression of key morphogenetic signals (Puelles et al., 2003; Puelles et al., 2004; Vernay et al., 2005; Bernard and Prochiantz, 2016; Sugiyama et al., 2009). Beyond its developmental role, *Otx2* regulates perineuronal net (PNN) activity, which modulates plasticity and closes sensitive periods for vocal learning in songbirds (Cornez et al., 2017). Our research has found that following brain injury, PNNs exhibit a brief period of increased plasticity that may facilitate recovery from injury (Talwalkar et al., 2024), however how changes in *Otx2* expression might trigger PNN formation and enhance neuronal plasticity remain unknown.

To begin investigating *Otx2*’s role following TBI, we examined its expression at two time points post-injury. The two timepoints selected have previously been identified as critical windows for steroid-mediated neuroprotection in the zebra finch brain, during which local estrogen synthesis particularly by reactive glia plays a key role in promoting neuronal survival and limiting apoptosis following injury (Duncan and Saldanha, 2011; Wynne et al., 2008; Pedersen et al., 2017). We observed a decrease in *Otx2* mRNA expression, suggesting a potential role in post-TBI recovery. Given the well-documented neuroprotective effects of estrogen in the zebra finch brain (Saldanha, 2020; Holloway and Clayton, 2001), we also tested whether estrogen availability influences injury-induced *Otx2* mRNA expression. These findings highlight *Otx2* as a promising candidate for future studies exploring its potential as a therapeutic target following damage to the brain.

## 2 Methods

### 2.1 Animals

Adult (> 90 days post hatching) Australian zebra finches were originally obtained from a breeder (Magnolia Bird Farms; Anaheim, CA) and group housed in the animal facility at Vassar College. The Vassar College Institutional Animal Care and Use Committee approved all animal procedures. All subjects were pseudo-randomly assigned to groups at the time of testing. Birds were provided with food and water *ad libitum* and maintained under a 14:10 light: dark cycle.

### 2.2 Penetrating brain injury

Prior to injury, 4% Lidocaine hydrochloride (Lidocaine Plus, Barboursville, WV) was applied to the top of the subject’s head. All subjects were then anesthetized with 5% isoflurane and maintained at 2–3.5% (Henry Schein Animal Health, Dublin, OH). Subjects were then positioned in a stereotaxic apparatus (Stoelting, Wood Dale, IL) with the head angled downward at 45°. When the cranium was exposed, a bore needle was used to create a bilateral craniotomy. The injury was targeted to the entopallial nucleus (2 mm anterior to the pineal, 3 mm lateral to the midline, 3 mm ventral to the dural surface) because this area lacks detectable steroid hormone/aromatase expression in the absence of injury in zebra finches (Wynne et al., 2008; Saldanha et al., 2000; Shen et al., 1995; Walters et al., 2011). A 22 s Hamilton syringe (Hamilton Company, Reno, NV) was positioned at the surface of the brain and lowered 3-mm to target the area. Subjects (*n* = 5–6 per treatment group, with equal males and females) were pseudo-randomly assigned to receive either Fadrozole or vehicle. The needle remained in position for 60 s. The scalp incision was sealed with Collodion Flexible (EM Science, Gibbstown, NJ) or Vetbond Tissue Adhesive (3 M, Saint Paul, MN) and birds were allowed to recover under a heat lamp until they were returned to the aviary. For sham animals, the cranium was exposed, but no craniotomy or injection was performed.

### 2.3 Fadrozole administration

Birds were administered either fadrozole (Sigma-Aldrich, St. Louis, MO, USA) or vehicle (saline) via intracranial injection at the time of surgery. Each bird received 5 μL of either fadrozole (10 mg/mL; 50 μg per injection) suspended in vehicle or 5 μL of vehicle alone. Injections were delivered at a rate of 2.5 μL/s (Wynne et al., 2008; Cook et al., 2019), and the needle was left in place for 90 s following infusion. This protocol has been previously shown to significantly reduce estrogen levels in brain tissue surrounding the injection site (Walters et al., 2011; Charlier et al., 2011; Pedersen et al., 2016).

### 2.4 cDNA synthesis and qPCR

Following an overdose of isoflurane, zebra finches were rapidly decapitated, and brain tissue was immediately extracted. One telencephalic lobe was dissected and homogenized in 1 mL of TRIzol reagent for RNA extraction. Individual birds and treatments were treated as biological replicates for all statistical analyses. Total RNA was isolated according to the manufacturer’s protocol. RNA quality and purity were assessed using a NanoDrop One spectrophotometer (NanoDrop, Wilmington, DE), and only samples with 260/280 absorbance ratios greater than 1.85 were included in downstream analyses. While 260/280 ratios are commonly used to evaluate RNA purity, we acknowledge that RNA Integrity Numbers (RIN) or gel electrophoresis were not performed, and this represents a limitation of the current study. For each qPCR experiment, 1 μg of total RNA was reverse transcribed using the High-Capacity cDNA Reverse Transcription Kit (Applied Biosystems). qPCR reactions were performed using 1 μL of resulting cDNA and SsoAdvanced Universal SYBR Green Supermix (Bio-Rad, Hercules, CA) in a total volume of 10 μL. Reactions were carried out in 96-well optical plates, with each sample run in duplicate on a QuantStudio 3 Real-Time PCR System (Thermo Fisher Scientific Inc.). Amplicons targeted exonic regions of the genes listed in Table 1.

**Table 1:** List of primers used for amplification for qPCR.

Gene	Accession number	Direction	Primer sequence
GAPDH control	XM_015873412.1	Sense antisense	TGC TGC TCA GAA CAT TAT CCC TTT CCC ACA GCC TAG CAG CT
*Otx2*	XM_030275214.2	Sense antisense	CCT CAG TCC CCA CCA TTT CC TCT CGT CGC GAT TTC TCT CG

### 2.5 Statistical analysis

The delta threshold cycle number (ΔCt) method was used for quantification of the qPCR data using the detection threshold (Ct) for each target gene less the Ct for the housekeeping gene, glyceraldehyde 3-phosphate dehydrogenase (GAPDH). GAPDH was chosen as a reference because of its stability as a reference gene in songbird brain and has been previously validated (Zinzow-Kramer et al., 2014; Rensel and Schlinger, 2020). Comparative Ct measurements give a relative expression difference between samples, where a lower number means greater mRNA expression. All statistical analyses were performed on ΔCt values, and are presented as means ± SEM. Data were analyzed using a two-way ANOVA with treatment and sex as between-subject variables in GraphPad Prism (GraphPad Software, Inc., La Jolla, CA). *Post hoc* comparisons following significant main effects or interactions were conducted using Sidak’s multiple comparisons test to control for Type I error. A significance level of *α* = 0.05 was used for all analyses.

## 3 Results

### 3.1 Experiment 1: *Otx2* expression following injury at 24 h and 1 week post injury

**Figure 1:**
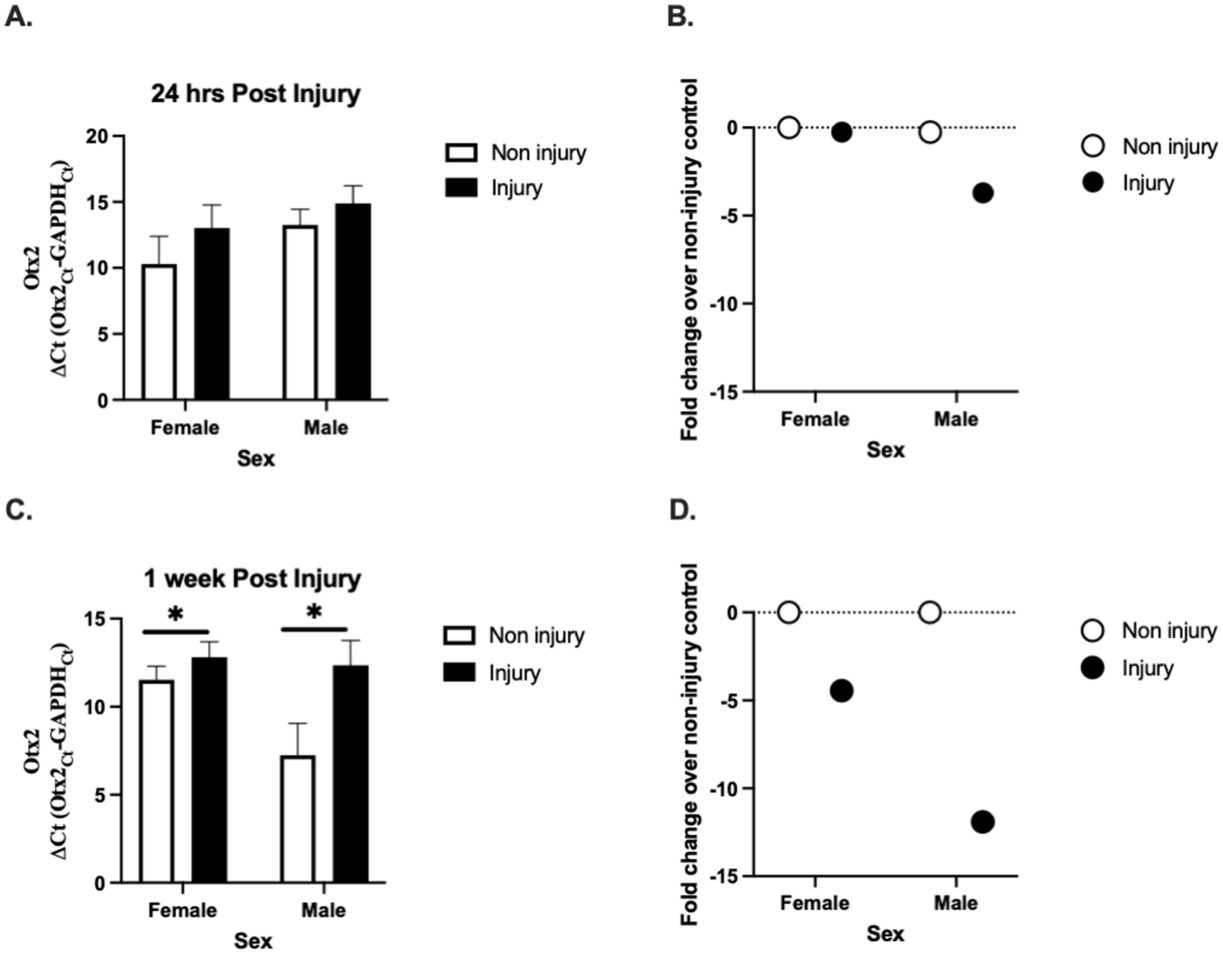
Effect of injury on *Otx2* mRNA expression in the adult zebra finch (ZF) brain. *Otx2* mRNA expression in adult male and female ZFs at 24 h **(A)** and 1 week **(C)** relative to GAPDH (ΔCt values ± SEM) following injury. Mean ΔCt values are presented where a lower number means greater mRNA expression. 1 week following injury *Otx2* mRNA expression was significantly lower compared to sham controls. There was no effect of sex at either timepoint. For a better clarity, fold change **(B,D)** values ± SEM over uninjured controls are also presented. The dashed line represents uninjured controls (controls were set to 1 for fold change calculation). *n* = 5 per sex and per treatment * Denotes a significant effect of injury at *p* < 0.05. These data suggest that Otx2 mRNA expression is decreased following injury and may allow for increased neuronal plasticity following injury **(B,D)**.

*Otx2* expression was measured at two timepoints (24 h (24 h) and 1 week(1 W)) following injury. Two-way ANOVA revealed a significant effect of injury on *Otx2* expression at 1 week (Figure 1C), but not for 24 h (Figure 1A); F_1W_(1, 15) = 6.847, *p* = 0.019; F_24h_(1, 15) = 0.192, *p* = 0.192, but not of sex F_1W_(1, 15) = 3.751, *p* = 0.072; F_24h_(1, 15) = 2.315, *p* = 0.149, and no overall interaction between sex and injury: F_1W_(1, 15) = 2.429, *p* = 0.140; F_24h_(1, 15) = 0.118, *p* = 0.735. These data suggest that *Otx2* mRNA expression is decreased following injury and may allow for increased neuronal plasticity following injury.

### 3.2 Experiment 2: homeodomain expression following injury and circulating estrogen depletion

**Figure 2:**
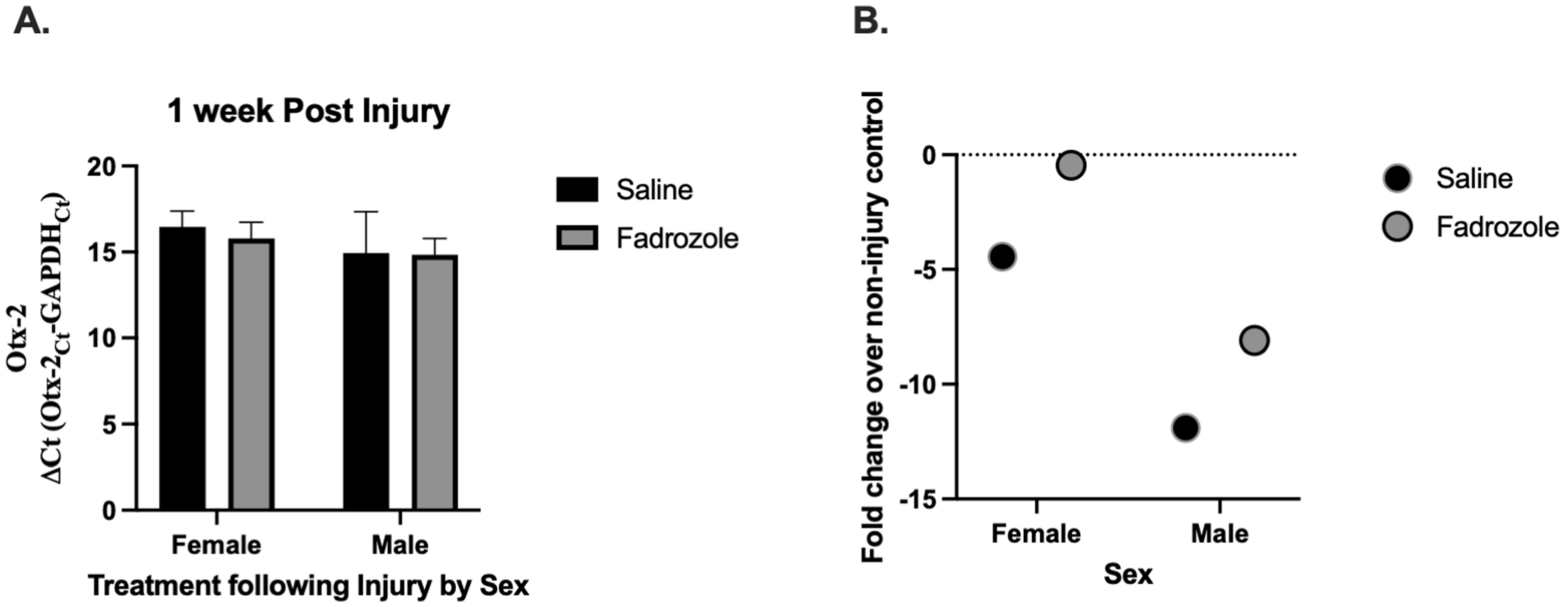
Effect of estrogen depletion on *Otx2* mRNA expression following injury. *Otx2* **(A)** mRNA expression relative to GAPDH (ΔCt values ± SEM) in adult male and female zebra finches at 1 week post injury. Fadrozole (aromatase inhibitor) treatment had no significant effect at 1 week. Mean ΔCt values are presented where a lower number means greater mRNA expression and fold change values. **(B)** Over injured controls are presented for clarity. The dashed line represents injured controls (controls were set to 1 for fold change calculation). *n* = 5–6 per sex and per treatment.

Following injury, *Otx2* mRNA expression (Figure 2A) was not altered by circulating estrogen levels; F(1, 20) = 0.07, *p* = 0.794 in males or females F(1, 20) = 0.729, *p* = 0.403 with no interaction between the two F(1, 20) = 0.04, *p* = 0.843. Fold change calculations show no difference in expression (Figure 2B).

## 4 Discussion

Our study examined the role of *Otx2* mRNA in the zebra finch brain following traumatic brain injury (TBI). We observed a significant decrease in *Otx2* mRNA expression post-injury (Figure 1A), suggesting an involvement in neuronal response to TBI and highlighting it as a potential target for further investigation. Despite the known neuroprotective effects of estrogens in the zebra finch brain, reduced estrogen levels had no effect on *Otx2* mRNA expression following injury. This suggests that *Otx2* mRNA regulation following TBI occurs independently of estrogen signaling.

### 4.1 *Otx2* and critical period plasticity

*Otx2* is a transcription factor essential for neuronal regulation and has been identified as a key regulator in closing critical periods of plasticity in both developing and adult brains (Sugiyama et al., 2009; Reichelt et al., 2019; Beurdeley et al., 2012). These critical periods represent windows of heightened neural plasticity during which environmental stimuli strongly shape circuit formation. Reopening such periods after injury may be important for enabling neural repair and reorganization (Hensch, 2005; Takesian and Hensch, 2013), a phenomenon also observed in mammalian models, suggesting an evolutionarily conserved mechanism (Fawcett et al., 2019; Pizzorusso et al., 2006).

While our interpretation is informed by *Otx2*’s established role in critical period closure (Sugiyama et al., 2009; Beurdeley et al., 2012; Spatazza et al., 2013), the transcriptional pathways involved may diverge following injury in the adult zebra finch brain (Spatazza et al., 2013; Di Nardo et al., 2018). Furthermore, we only examined *Otx2* mRNA expression at two timepoints. These timepoints represented pivotal times associated with estrogen mediated neuroprotection, but later timepoints may be necessary to fully understand the role of *Otx2* in neuronal recovery. Future studies incorporating functional and behavioral analyses are necessary to clarify the role of *Otx2* in injury-induced neuroplasticity and critical periods. It is important to note that mRNA expression does not always correlate with protein abundance due to post-transcriptional regulation, mRNA stability, and translational control; thus, without protein-level validation, the observed downregulation of *Otx2* mRNA remains an incomplete indicator of functional change. Notably, *Otx2* mRNA expression was unaffected by estrogen depletion, suggesting that its regulation may occur independently of estrogen signaling in this context. Estrogens are known to influence the growth and connectivity of song nuclei during the critical period, increasing HVC volume and promoting synaptogenesis (Fusani and Gahr, 2006; Herrmann and Arnold, 1991; Charlier et al., 2010; Brenowitz, 2015). After injury, local estrogen production is upregulated through aromatase-expressing glial cells, a response that supports neuronal survival and repair (Saldanha, 2020; Lee et al., 2007; Duncan, 2020). Further research incorporating functional, behavioral, and hormonal measures will be necessary to determine whether *Otx2* downregulation supports or impairs recovery after TBI.

## 5 Conclusion

Our findings highlight Otx2 as potential target for further study into the mechanisms and therapeutic strategies surrounding neuronal injury. Future research should focus on elucidating the molecular mechanisms underlying this process and exploring therapeutic strategies that harness critical period plasticity to promote recovery from neuronal injuries (Hensch, 2005; Bavelier et al., 2010). By drawing on developmental parallels, we may be able to design interventions that optimize recovery outcomes and improve the quality of life for individuals with brain injuries.

## Data Availability

The raw data supporting the conclusions of this article will be made available by the authors, without undue reservation.
